# Single-Center Experience With the Thoraflex™ Hybrid Prosthesis: Indications, Implantation Technique and Results

**DOI:** 10.3389/fcvm.2022.924838

**Published:** 2022-05-30

**Authors:** Konrad Wisniewski, Arash Motekallemi, Angelo M. Dell'Aquila, Alexander Oberhuber, Johannes F. Schaefers, Abdulhakim Ibrahim, Sven Martens, Andreas Rukosujew

**Affiliations:** ^1^Department of Cardiothoracic Surgery, University Hospital Muenster, Muenster, Germany; ^2^Department of Vascular and Endovascular Surgery, University Hospital Muenster, Muenster, Germany

**Keywords:** frozen elephant trunk (FET), Thoraflex Hybrid prosthesis, acute aortic dissection (AAD), thoracic aortic aneurysm (TAA), post-dissection aneurysm

## Abstract

**Objective:**

The aim of this study was to evaluate the early and mid-term results after the frozen elephant trunk (FET) procedure for the treatment of complex arch and proximal descending aortic disease in a single-center institution.

**Methods:**

From April 2015 to July 2021, 72 patients (25 women, 60.4 ± 10.3 years) underwent Thoraflex™ Hybrid implantation at our institution. The indications were thoracic aortic aneurysm (TAA) (*n* = 16, 22.2%), post-dissection aneurysm (*n* = 21, 29.2%), and acute aortic dissection (AAD) (*n* = 35, 48.6%). Antegrade cerebral perfusion under moderate hypothermia (28°C) was employed in all cases. Eighteen patients (25%) have already been operated due to heart or aortic disease.

**Results:**

Overall in-hospital mortality was 12.5% (9 patients). Rates of permanent neurological dysfunction and spinal cord injury were 9.7 and 5.5%, respectively. The in-hospital mortality rate among patients operated on AAD, TAA, and post-dissection aneurysm were 8.6, 6.2, and 23.8%, respectively. At a mean follow-up of 26 ± 20 months, mortality was 9.7%. Furthermore, 23 patients (31.9%) required a subsequent procedure in distal aorta: endovascular stentgraft extension in 19 patients (26.4%) and open aortic surgery in 4 patients (5.5%). The mid-term survival of patients with type A aortic dissection was 97%.

**Conclusions:**

Our experience with the Thoraflex Hybrid prosthesis demonstrates its surgical applicability for different types of aortic pathologies with promising outcomes during early and midterm follow-up. Our technique and perioperative management lead to comparable or even superior neurological outcomes and mortality in urgent cases considering other high-volume centers.

## Introduction

The Thoraflex™ Hybrid prosthesis was first introduced in 2010 (1) for the treatment of combined diseases of the aortic arch and the proximal descending aorta (2).

Since then, the application of Thoraflex™ Hybrid prosthesis with two modifications (Thoraflex™ Plexus 4^®^ and Ante Flo^®^, Vascutek, Terumo, Inchinnan, Scotland, UK) has become a widely established approach for the treatment of acute and post-dissection aneurysms as well as aneurysms of the thoracic aorta (thoracic aortic aneurysm, TAA) (3–5).

With the addition of the latest model, the frozen elephant trunk (FET), the surgeons' armamentarium has grown significantly in recent years and hybrid prostheses have become applicable to a wider field of indications. The 4-branched Thoraflex™ hybrid graft and its counterparts, such as the Jotec E-vita NEO (Jotec GmbH, Hechingen, Germany), not only enable a distinctive surgical approach (in case of dissection) of the supra-aortic vessels but also the endoluminal treatment of the proximal descending aorta. Thus, the FET technique not only affects the stent-grafted segment but also the downstream aorta with positive effects on aortic remodeling. It also enables subsequent procedures, either one or second stage (i.e., endovascular stent extension or open aortic repair).

Various groups (6–8) demonstrated the benefits of the hybrid stentgraft technique on true lumen collapse avoidance and aortic remodeling, besides other benefits (i.e., lower body malperfusion, mortality and secondary aortic intervention, and neurological complications) (4, 9–12).

At our institution, the first Thoraflex™ Hybrid prosthesis was implanted in April 2015. This study aimed to present our single-center experience regarding the indications, implantation technique, as well as the perioperative and mid-term results.

## Materials and Methods

### Patients

From April 2015 to July 2021, 72 patients (25 women, 60.4 ± 10.3 years) with pathologies of the ascending aorta and aortic arch underwent surgical treatment with the Thoraflex™ Hybrid prosthesis (*Terumo Aortic, Vascutek Ltd., Inchinnan, UK)* at our department. The ethical committee of our institution approved this retrospective study (2020-126-f-S). Data from the perioperative patients were reviewed retrospectively. The follow-up data regarding survival were retrieved from the residents' registration offices. The follow-up was completed in October 2021. The data were analyzed according to the underlying aortic disorder (aneurysm, acute dissection, and post-dissection aneurysm). Patients' demographics are presented in Table 1. Coronary angiography, echocardiography, and computed tomography angiography (CTA) were routinely performed for all elective cases. In patients with acute aortic dissection (AAD), the diagnosis was confirmed by (ECG-triggered) CTA. The same surgical team performed all cases.

**Table 1:** Demographic and pre-operative clinical data.

	**All—*n*/mean (IQR/%/SD)**	**Acute dissection, *N* = 35**	**Post-dissection aneurysm, *N* = 21**	**Aortic aneurysm, *N* = 16**	***P*-value**
Age (years)	60.4 (61)	57.3 (56)	59.2 (60)	68.7 (70)	**<0.0001**
Sex (female)	25 (34.7%)	7 (20%)	8 (38.1%)	10 (62.5%)	0.013
Body Mass Index	26.7 (26)	27.0 (27)	27.5 (26)	25.1 (23.5)	0.21
History of smoking	22 (30.5%)	6 (17.1%)	6 (28.6%)	10 (62.5%)	**0.006**
Diabetes mellitus	4 (5.6%)	0 (0%)	3 (14.3%)	1 (6.2%)	0.058
Dyslipidemia	18 (25.0%)	3 (8.6%)	7 (33.3%)	8 (50.0%)	**0.003**
Arterial hypertension	53 (73.6%)	22 (62.8%)	19 (90.5%)	12 (75.0%)	0.078
Peripheral vascular disease	3 (4.2%)	0 (0%)	1 (4.8%)	2 (12.5%)	0.074
Cerebrovascular disease	5 (6.9%)	2 (5.7%)	2 (9.5%)	1 (6.25%)	0.84
Abdominal aortic aneurysm	15 (21.0%)	1 (2.8%)	6 (28.6%)	8 (50.0%)	**0.0001**
Pulmonary disease (COPD)	9 (12.5%)	1 (2.8%)	2 (9.5%)	6 (37.5%)	**0.003**
Connective tissue disorder	5 (6.9%)	1 (2.8%)	4 (19.0%)	0 (0%)	0.05
Pre-operative history of stroke	9 (12.5%)	2 (6.7%)	4 (19.0%)	3 (18.75%)	0.19
New-onset cerebral ischemia	4 (5.6%)	3 (8.6%)	0 (0%)	1 (6.25%)	0.44
Pre-operative paraplegia	4 (5.6%)	4 (11.4%)	0 (0%)	0 (0%)	0.18
Pre-operative atrial fibrillation	6 (8.3%)	2 (5.7%)	1 (4.8%)	3 (18.75%)	0.34
Previous surgery	18 (25.0%)	1 (2.8%)	16 (79.2%)	1 (6.2%)	**<0.0001**

*Statistically significant values are in bold*.

### Surgical Technique and Perioperative Management

Standard access for complex aortic lesions at our institution is median sternotomy. Arterial cannulation was performed directly through the right axillary artery and venous cannulation through the right atrium in most cases. The left side of the heart was vented through the right superior pulmonary vein. In selected cases, permanent antegrade perfusion of the heart made it possible to perform an aortic prosthetic repair on a beating heart. The carbon dioxide insufflation was administered in the operative field to minimize the risk of air embolism. Cold blood cardioplegia was our first choice for myocardial protection and was administered mostly retrograde through the coronary sinus or directly into the coronary ostia.

Replacement of the aortic arch was performed under moderate hypothermic (28°C on average) circulatory arrest and selective antegrade cerebral perfusion (SACP) with the application of near-infrared spectroscopy (NIRS). Initially, unilateral SACP with permanent monitoring of cerebral tissue oxygenation was performed. If needed, the conversion from unilateral perfusion to bilateral was performed through direct cannulation of the left common carotid artery. After reaching the desired temperature and circulatory arrest, the aortic arch in Zone 2 or 3 was transected distally and the endoprosthesis was deployed into the descending aorta. A sewing collar simplifies and reinforces the distal anastomosis. Additional sealing is achieved with (Teflon) felt strips and the application of the two-component BioGlue^®^ Surgical Adhesive (CryoLife, Inc.) on the suture line. After completion of the distal anastomosis, lower body reperfusion is obtained *via* the extracorporeal circulation branch of the graft.

**Figure 1:**
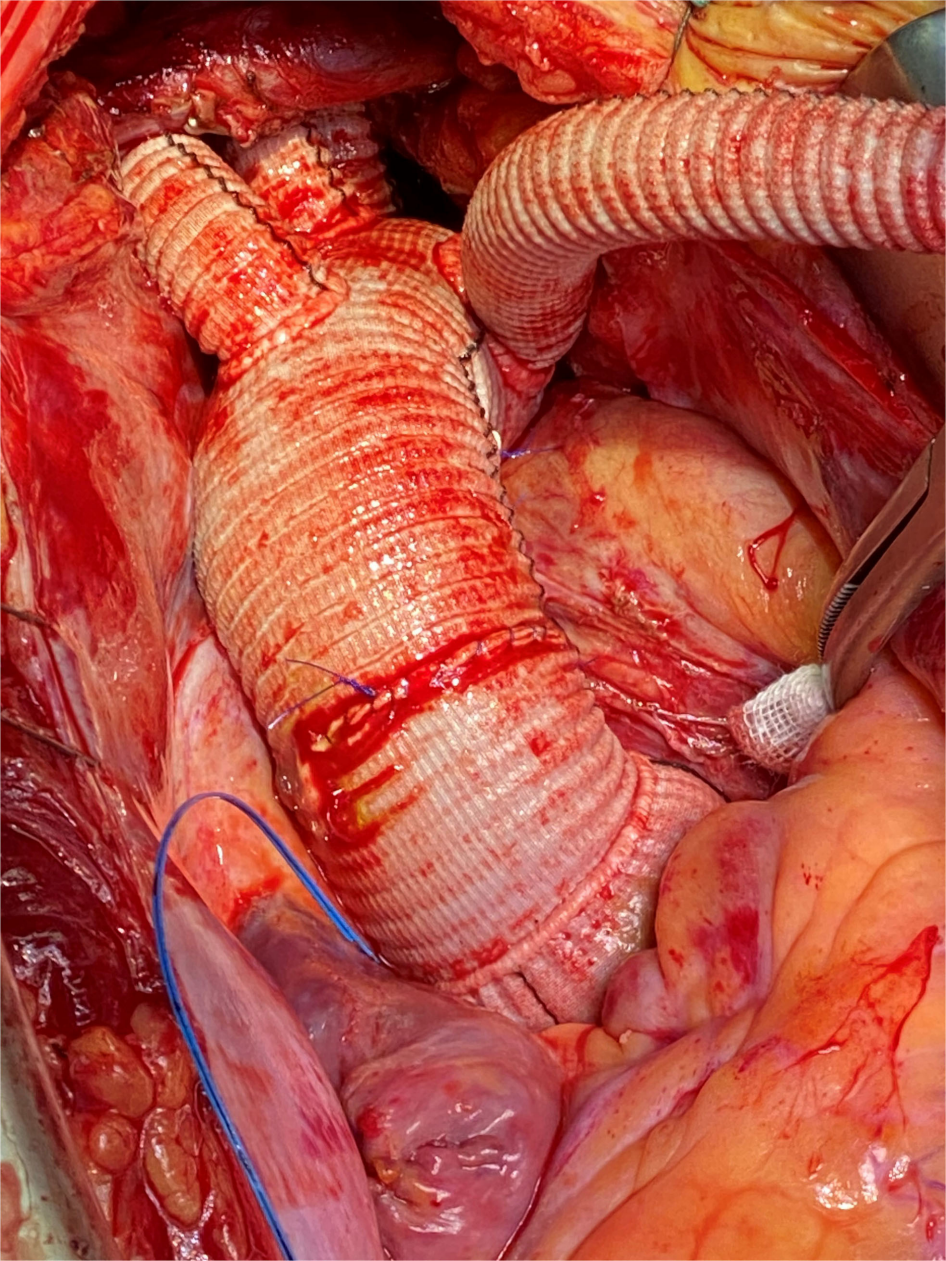
Intraoperative photography, complete implantation of the Thoraflex Hybrid prosthesis with mechanical conduit.

To keep cardiac arrest to a bare minimum, cardiac perfusion is reestablished after repair of the subclavian artery and/or proximal anastomosis of the ascending aorta in the case of supracoronary replacement. The remaining vessels are then anastomosed after clamp removal. In the case of the Bentall procedure, we perform aortic root replacement after anastomosing all supracoronary vessels. After de-airing the heart, the coronary circulation was reinitialized (Figure 1). Concomitant procedures, e.g., mitral valve replacement and coronary artery bypass grafting, were typically performed after the repair of the left subclavian artery and after the reestablishment of lower body perfusion. Under normothermia, the cardiopulmonary bypass (CPB) was terminated, and the fourth graft branch, used for antegrade perfusion, was ligated and resected.

The perioperative management is standardized for procedures in our center. Prophylactic cerebrospinal fluid (CSF) drainage was not perioperatively performed. All patients with diagnosed spinal ischemia received post-operative CSF drainage and MRI. To reduce the duration of hypothermic circulatory arrest (HCA), rewarming and perfusion of the lower body were restarted after completion of the distal aortic arch anastomosis. Patients received CT angiograms (CTA) from the neck vessels to the iliac or femoral arteries routinely before discharge. In the case of suspected ischemia, CTA is performed immediately after the procedure.

An important component of anesthesiologic support was the prevention of excessive blood loss in the early post-operative stage. Before intervention, the patient was given a fibrinolysis inhibitor—tranexamic acid as a short infusion, then continuously during the intervention. For optimal assessment of the blood coagulation, during the weaning of the patient of the CPB, the thromboelastography was performed simultaneously with activated clotting time control after administration of protamine sulfate.

### Statistical Analysis

The statistical analysis was accomplished with Statistica 13.3 (StatSoft, TIBCO Software, Palo Alto, CA, USA) for Windows. Continuous variables were presented as mean ± standard deviation (SD) or median and interquartile range (IQR). Categorical variables were given as total numbers and percentages. Fisher's exact test and its Freeman–Halton extension were used to analyze differences in dichotomous variables. The non-parametric Mann–Whitney *U*-test was used for group comparisons of continuous variables. The Kruskal–Wallis test was used for multiple group comparisons of continuous variables. The Kaplan–Meier survival estimate was used for survival analysis. Statistical differences in Kaplan-Meier survival were determined with the log-rank test.

## Results

The detailed intraoperative and the post-operative data are presented in Tables 2, 3, respectively. Overall, this study population showed a high operative risk, and 35 cases (48.6%) had an AAD: 29 patients had type A dissection (acute aortic dissection type A, AADA) and 6 with type B dissection (acute aortic dissection type B, AADB). There were 16 cases (22.2%) with TAA and 21 patients (29.2%) were diagnosed with post-dissection aortic aneurysms (PDAAs). Arterial cannulation through the right axillary artery was performed in 98.6% of our cases. In two cases, femoral artery cannulation was needed, in one case due to local dissection of the axillary artery and another case due to reoperation and adhesions. The Thoraflex Hybrid prosthesis was applied successfully in all cases. In our investigated cohort, patients with aortic aneurysms were significantly older (*p* < 0.0001) than patients with acute and post-dissection aneurysm. In all groups, patients were predominantly men. Patients with aneurysm had shorter operation times and were more often accompanied by concomitant procedures (3 cases with coronary artery bypass graft [CABG]). Contrastingly, post-dissection aneurysm treatment was significantly longer (*p* < 0.0001). The mean hospital stay of the whole cohort lasted 16 days. Patients with AAD patients had a prolonged intensive care unit (ICU) stay and overall longer hospital stay, 9.7 and 19.5 days, respectively (*p* < 0.12). The mean ventilation time in the entire cohort was 24 h and was significantly shorter (<1 day) in elective cases. Only patients with AAD required ventilation of ~33 h. Furthermore, 17 patients (23.6%) required temporary hemofiltration due to acute kidney injury (9 were patients with AAD). Only one patient required permanent dialysis after discharge.

**Table 2:** Intraoperative data.

	**All—*n*/mean (IQR/%/SD)**	**Acute dissection, *N* = 35**	**Post-dissection aneurysm, *N* = 21**	**Aortic aneurysm, *N* = 16**	***P*-value**
Duration of surgery (min.)	345.7 (±78)	338.9 (±65)	390.0 (±95)	302.2 (±51)	**0.007**
CPB time (min.)	221.3 (±62)	215.9 (±41)	252.0 (±85)	192.7 (±47)	**0.007**
Cross clamp time (min.)	133.4 (±55)	139.4 (±54)	133.6 (±70)	120.2 (±34)	0.46
Distal HCA time (min.)	46.2 (±11)	48.3 (±9)	46.2 (±13)	41.5 (±11)	0.18
HCA temperature (°C)	28.0 (±1.2)	27.9 (±1.5)	28.2 (±1.2)	27.9 (±0.3)	0.74
Supracoronary AAR	60 (83.3%)	27 (77.1%)	17 (80.9%)	16 (100%)	0.096
Bentall with biological conduit	9 (12.5%)	5 (14.3%)	4 (19.0%)	0 (0%)	0.17
Bentall with mechanical conduit	3 (4.2%)	3 (8.5%)	0 (0%)	0 (0%)	0.31
Additional mitral valve replacement	1 (1.39%)	0 (0%)	1 (4.8%)	0 (0%)	0.51
Additional CABG	7 (9.7%)	2 (5.7%)	2 (9.5%)	3 (18.7%)	0.39
Prosthesis diameter (mm)	26 (24–30)	26 (24–26)	26 (26–30)	30 (30–30.5)	**<0.0001**
Stent diameter (mm)	28 (26–32)	28 (26–28)	28 (26–32)	34 (32–36.5)	**<0.0001**
Stent length-−150 mm	23 (31.9%)	14 (40.0%)	4 (19.0%)	5 (31.2%)	0.29
Distal anastomosis in Zone 0	1 (1.4%)	1 (2.8%)	0 (0%)	0 (0%)	1.0
Distal anastomosis in Zone 1	0 (0%)	0 (0%)	0 (0%)	0 (0%)	1.0
Distal anastomosis in Zone 2	21 (29.2%)	9 (25.7%)	8 (38.1%)	4 (25.0%)	0.59
Distal anastomosis in Zone 3	50 (69.4%)	25 (71.4%)	13 (61.9%)	12 (75.0%)	0.71

*CPB, Cardiopulmonary bypass; HCA, Hypothermic circulatory arrest; AAR, Ascending aortic replacement; CABG, Coronary artery bypass graft. Statistically significant values are in bold*.

**Table 3:** Post-operative data.

	**All—*n*/mean (IQR/%/SD)**	**Acute dissection, *N* = 35**	**Post-dissection aneurysm, *N* = 21**	**Aortic aneurysm, *N* = 16**	***P*-value**
Inhospital mortality	9 (12.5%)	3 (8.6%)	5 (23.8%)	1 (6.2%)	**0.04**
Length of ICU stay (d)	7.9 (3–9)	9.7 (3–11)	4.8 (3–5)	8.2 (3–11.5)	0.12
Length of stay (d)	15.7 (10–19)	19.5 (13–26)	11.5 (8–15)	13.5 (10.7–15.2)	**<0.0001**
Tracheostomy	6 (8.3%)	5 (14.3%)	0 (0%)	1 (6.2%)	0.17
Reintubation	9 (12.5%)	5 (14.3%)	2 (9.5%)	2 (12.5%)	0.9
Ventilation time (h)	24.2 (±45)	33.3 (±62)	15.7 (±16)	15.6 (±17)	0.31
Myocardial infarction	1 (1.4%)	1 (2.8%)	0 (0%)	0 (0%)	1.0
Re-exploration for bleeding	8 (11.1%)	4 (11.4%)	3 (14.3%)	1 (6.2%)	0.89
ECLS	5 (6.9%)	0 (0%)	3 (14.3%)	2 (12.5%)	0.036
post-operative CPR	7 (9.7%)	3 (8.6%)	3 (14.3%)	1 (6.2%)	0.77
Permanent neurological deficit	7 (9.7%)	4 (11.4%)	3 (14.3%)	0 (0%)	0.35
Paraparesis	4 (5.5%)	3 (8.6%)	1 (4.8%)	0 (0%)	0.81
Recurrent nerve palsy	5 (6.9%)	2 (5.7%)	1 (4.8%)	2 (12.5%)	0.70
Mesenteric ischemia	5 (6.9%)	2 (5.7%)	2 (9.5%)	1 (6.2%)	0.83
Atrial fibrillation	8 (11.1%)	5 (14.3%)	0 (0%)	3 (18.7%)	0.10
Temporary Hemofiltration	19 (26.4%)	9 (25.7%)	7 (33.3%)	3 (18.7%)	0.61
Presternal wound infection	2 (2.7%)	1 (2.8%)	1 (4.8%)	0 (0%)	1.0

*ICU, Intensive care unit; ECLS, Extracorporeal Life Support; CPR, Cardiopulmonary resuscitation. Statistically significant values are in bold*.

Four patients (5.5%) suffered from paraparesis, three of them were patients with AAD where the stentgraft was anastomosed in Zone 3 but only one of them received a 150 mm stentgraft. Paresis of the recurrent laryngeal nerve occurred in 5 cases (6.9%), mainly in patients with aortic aneurysm procedures. Permanent neurological deficit occurred in 7 cases (9.7%). We experienced only two cases of wound infection. One case of myocardial infarction after acute dissection was observed. Overall 5 patients (6.9%) suffered from mesenteric ischemia. In 8 cases (11%), atrial fibrillation occurred post-operatively. Cardiopulmonary reanimation was necessary in 7 cases (9.7%), three of them (14.3%) were post-dissection aneurysm patients. Extracorporeal life support (ECLS) was necessary in none of the acute dissection cases, while patients with post-dissection aneurysms and degenerative aneurysms were supported in 3 (14.3%) and 2 (12.5%) cases, respectively. Surgical re-exploration because of bleeding was needed in 8 cases (11%).

Early mortality expressed as in-hospital mortality was 12.5% (9 perioperative deaths) in the whole population. In acute cases of aortic dissection, only 3 patients died (8.6%, due to multiple-organ failures and cardiac tamponade). After elective aneurysm operation, we observed only 1 lethal case (6.2%, due to mesenteric ischemia) and 5 in-hospital deaths after post-dissection aneurysm procedure, reaching relatively high mortality of 23.8% (due to excessive bleedings, mesenteric ischemia with multiple-organ failure, and heart failure with cerebral ischemia).

**Figure 2:**
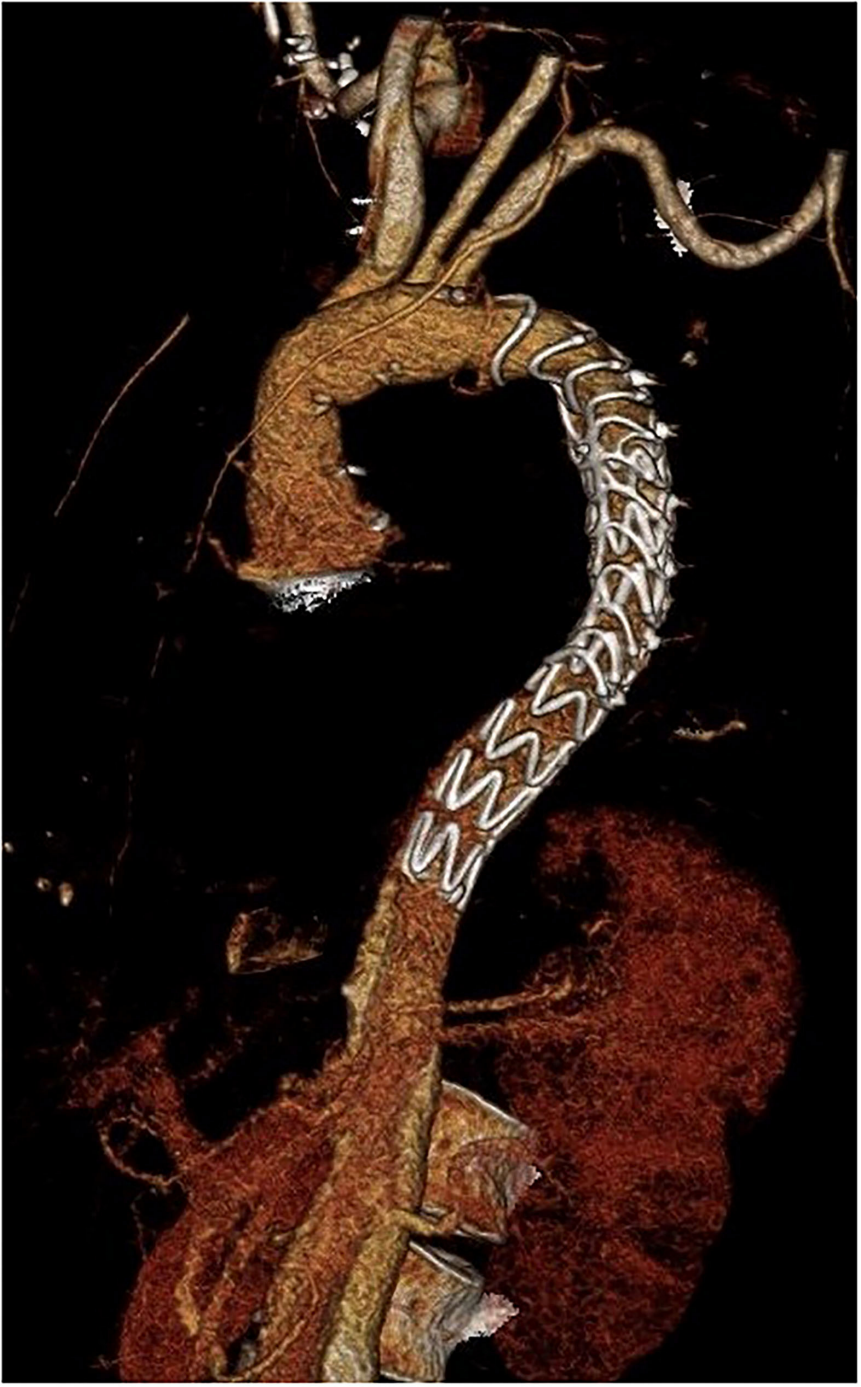
Three-dimensional computer tomography angiography (CTA) after distal stentgraft extension succeeding after implantation of the Thoraflex Hybrid prosthesis.

**Figure 3:**
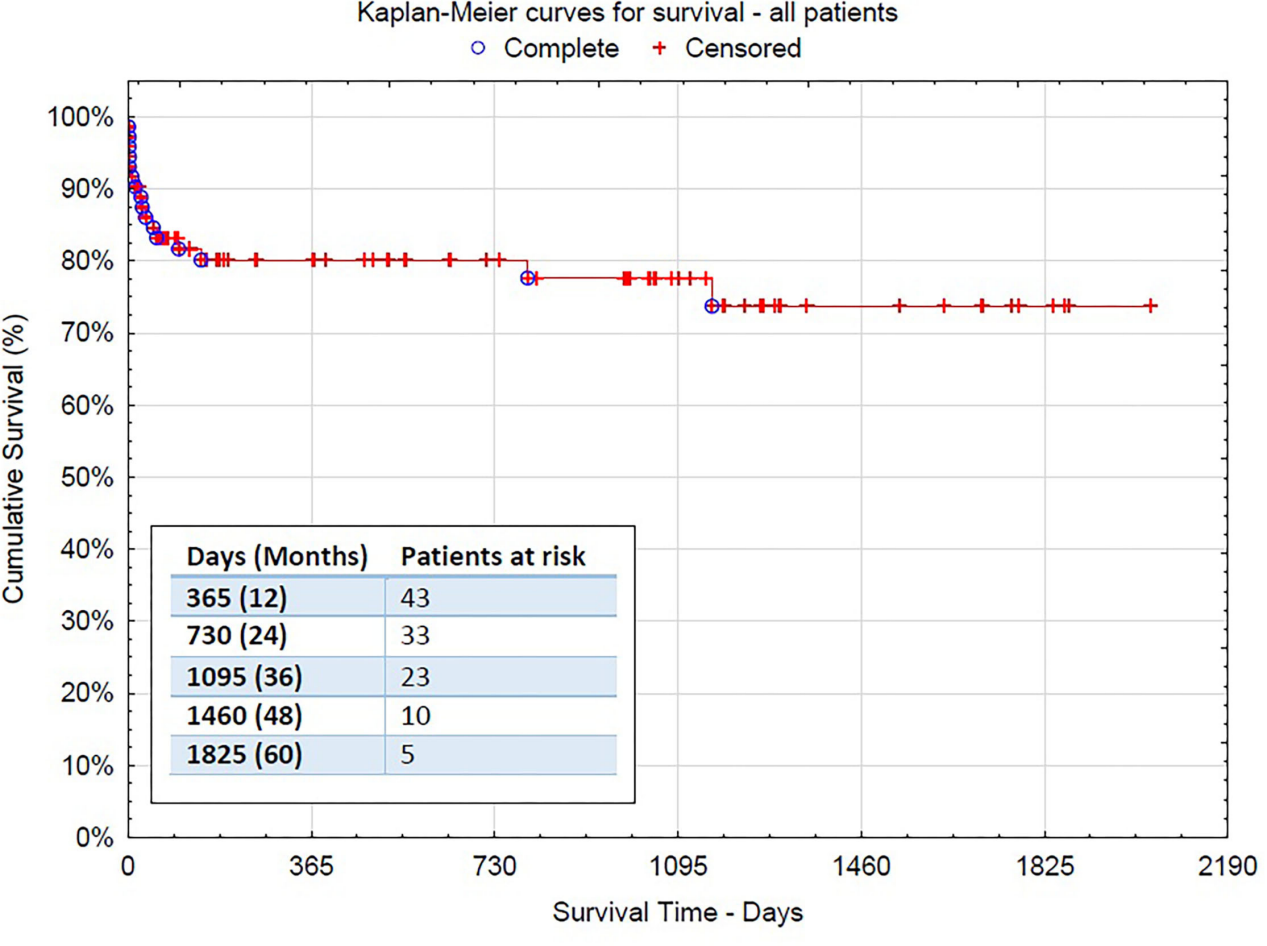
The Kaplan–Meier survival curve for the overall surgical population−95% confidence interval [*CI*] 1,574.015 (1,374.679–1,773.351).

**Figure 4:**
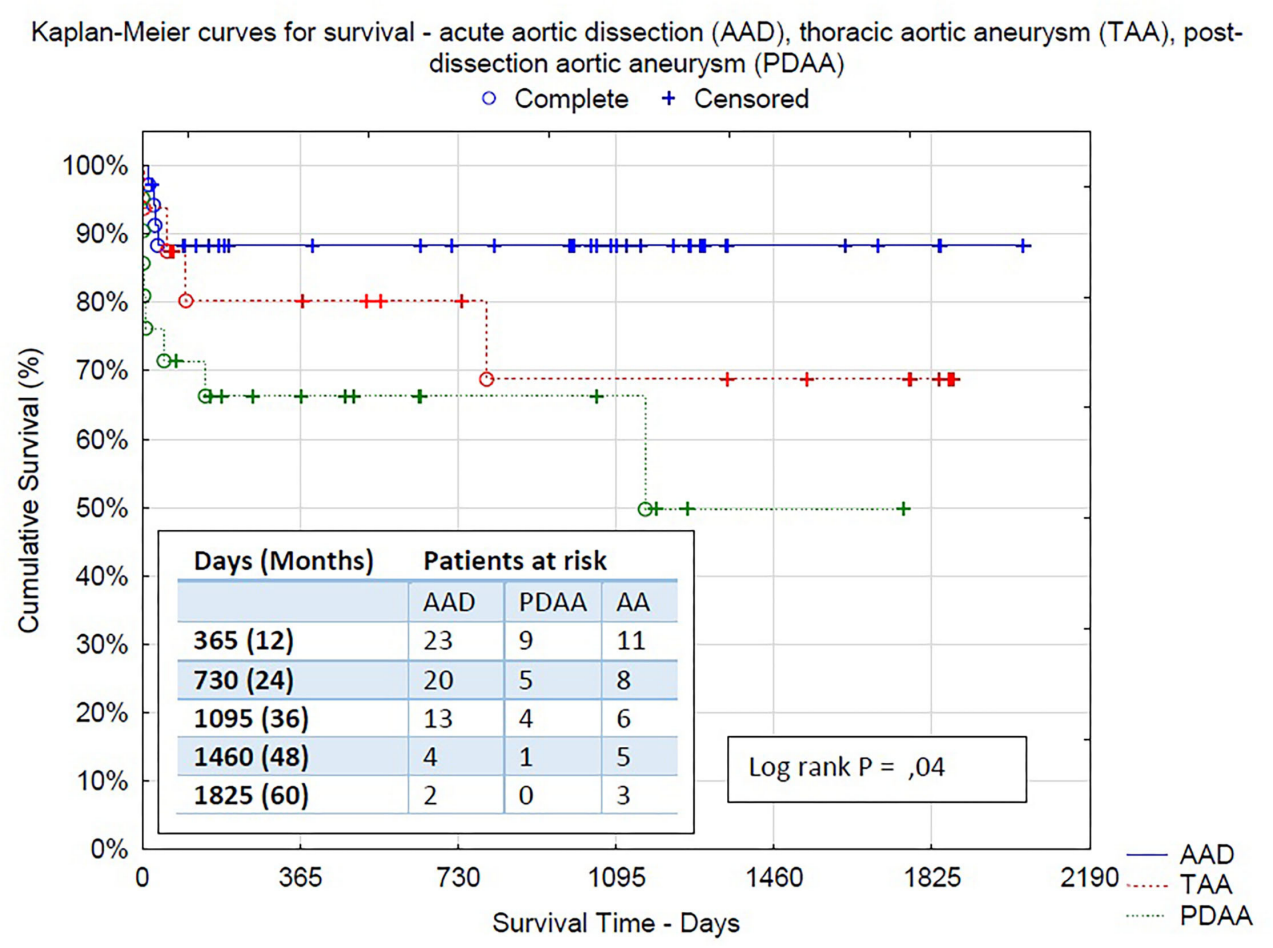
Kaplan–Meier survival curves between all three groups: acute aortic dissection (AAD), thoracic aortic aneurysm (TAA), post-dissection aortic aneurysm (PDAA)−95% *CI* 1,801.015 (1,585.471–2,018.503), 95% *CI* 1,390.010 (986.730–1,793.290), 95% *CI* 1,574.015 (1,374.679–1,773.351), respectively.

After a mean follow-up of 2 years, the mid-term mortality of patients was 9.7% (7 deaths-−3 due to aortic rupture, 3 due to infection or pneumonia, and 1 heart failure). All acute dissection patients but one survived after discharge and are doing well until the end of follow-up (2.8% mortality after discharge). In total, 19 patients (26.4%) received additional endovascular treatment with stentgraft implantation (thoracic endovascular aneurysm repair [TEVAR]) in the descending aorta (3 thoracoabdominal aortic aneurysm [TAAA], 9 PDAA, and 7 AAD) (Figure 2). Nine of them were performed during the same hospital stay after the FET procedure. Only in 4 cases, open surgical repair was needed due to the complexity of dissection in the thoracoabdominal aorta. Kaplan–Meier curves for survival are shown in Figures 3, 4.

## Discussion

The Thoraflex Hybrid graft combines the advantages of the FET technique with the aortic arch treatment, such as supra-aortic vessel repair. Our institutional experience since 2015, including the treatment of 72 patients with the Thoraflex Hybrid prosthesis, demonstrates its surgical applicability for different types of aortic pathologies with excellent outcomes during early and midterm follow-up. Our study showed that particular properties of our surgical technique (i.e., right subclavian artery cannulation, SACP, and moderate hypothermia) and the standardized perioperative management lead to comparable or even superior outcomes about neurological complications and mortality in urgent cases considering other high volume centers (3, 4, 6, 9, 11, 12). Interestingly, in patients with type A aortic dissection, in-hospital mortality was seen in only 8.6% of all cases, whereas mid-term survival at 26 months of discharged survivors was 97%. This is especially surprising with regard to the urgent nature of the disease.

Only in two cases, we use the Thoraflex Ante Flo with the “island technique” because of practical and technical issues. The use of a single branched graft facilitates suturing of the supraaortic vessels to the graft. Acute dissection membrane often extends to the left subclavian artery and requires proximal resection followed by a separate connection with a vascular interposition graft. The anastomosis of the brachiocephalic trunk and left common carotid artery in the form of a vascular island may be performed in an open fashion. This increases the duration of circulatory arrest, but this is technically more difficult due to the rigidity of the straightened stentgraft and antegrade perfusion branch, located in the same place. In our opinion, the Plexus version hybrid graft has a series of technical advantages, allowing better bleeding control of cranial vessels anastomosis, enabling partial clamping of the aorta, and complete removal of dissected tissue.

The Thoraflex stent size and length were determined pre-operatively according to the underlying disease and to total aortic diameter at the landing zone in patients with aneurysm or to the true lumen diameter in cases with acute dissection and post-dissection aneurysms. We avoided undersizing in dissected patients. In aneurysm, we performed a slight oversizing, ~15% of the distal landing zone. The diameter of the FET is generally a compromise between the diameter of the true lumen and the total aortic diameter.

We prefer extra-thoracic antegrade arterial cannulation *via* the right subclavian or axillary artery. This ensures better perfusion of cerebral vessels and simplifies SACP, providing technical freedom in the prosthetic repair of the aortic arch. The arterial cannulation through the right subclavian artery has been proven to improve neurologic results (13). Compared with the direct aortic cannulation in patients with the aortic arch disease, cannulation of the right subclavian artery is an easily applicable and efficient method for perioperative cerebral protection, facilitating SACP (13, 14).

Although technical issues might be associated with extra-thoracic cannulation (i.e., injury of the right brachial plexus), our institutional experience does not show major disadvantages of this cannulation technique. Our comparatively good results might be partly explained by the experienced surgeons who perform aortic surgery in our center. On the other hand, a major disadvantage of the direct aortic cannulation technique is the uncertainty regarding secure cerebral perfusion. In terms of the optimal arterial cannulation technique, it generally depends on the patient's hemodynamic condition, surgeon's experience, and anatomical variability. In urgent conditions, this is impossible to perform, as it is time consuming and a technically demanding method of cannulation.

In this technique of an SACP, mentioned in previous studies (13, 15), placement of a balloon catheter and manipulations of the carotid arteries are avoided, and reducing the chance for thromboembolic complications and/ or air embolism (13). Brain protection methods were well-assessed and proved as well as risk factors for permanent neurological injury, e.g., acute type A dissection and duration of circulatory arrest (15).

The advantages of unilateral and bilateral perfusion are still being discussed. Some teams are proving that results for both strategies are comparable, but the bilateral antegrade perfusion allows longer circulatory arrest (16, 17). Tsagakis et al. proposed four-sites perfusion achieving favorable results (18). Although very promising results were achieved with this sophisticated method, our institutional experience shows comparable results in a less complex approach.

In our cohort, permanent neurological deficits were found in 7 cases (9.7%). We believe there is still a place for improvement to minimize this detrimental complication. We do not perform pre-operative spinal cord protection as Shrestha and colleges do (6). In our experience, the percentage of paraplegic complication was lower than what they described (7 vs. 5.5% in our cohort). To the best of our knowledge, there is no sufficient evidence to recommend the usage of prophylactic CSF—monitoring/drainage, but more studies are necessary (5). Distal anastomosis occurred in 69.4% of cases (50 patients) in Zone 3 (distal to left subclavian artery), and only 29.2% (21 patients) in Zone 2. Majority of Zone 3 were cases of AAD (25 patients, 71.4% in this group).

Current options and recommendations for the treatment of thoracic aortic pathologies involving the aortic arch from the European Association for Cardio-Thoracic Surgery and the European Society for Vascular Surgery advise “proximalization” of the distal anastomosis from Zone 3 to 2 to shorten lower body circulatory arrest (5). In our experience, aortic dissection membrane often reaches into the left subclavian artery (secondary intimal ruptures localized in Zone 3) and descending part of the aorta. Unfortunately, this can be associated with a higher rate of laryngeal nerve palsy, which we observed in 3 cases (2 cases in Zone 2 deployment, overall 5 cases, 6.9%). Therefore, some authors recommend Zone 2 as the destination for the distal anastomosis (19).

In our latest experience, the avoidance of cardiac arrest may provide beneficial effect for patients' recovery as Martens et al. have shown in their protocol (20). In the case of retrograde AAD, where the dissection membrane does not spread extent proximally to the brachiocephalic trunk, it is also possible to perform aortic prosthetic repair as a beating heart procedure. Continuous perfusion of the heart is provided by a needle vent catheter used for antegrade perfusion and cooled to 32–34°C. The aorta is double-clamped in its proximal and distal parts and then transected to perform reconstruction on the aortic arch.

In patients with post-dissection aneurysm in our population, we observe relatively high mortality rates. The mean surgery time in this group was also the longest in our cohort due to the complexity of this pathology. Almost 80% of patients had undergone previous surgery before, and the majority of them suffered from multiple comorbidities. A relatively high rate of postprocedural exploration because of bleeding, neurological deficits, and mesenteric ischemia resulted in poorer results. In this group post-operatively cardiopulmonary reanimation or eventually ECLS was needed, resulting also in higher neurological deficits rates. However, these findings were not statistically significant due to relatively small numbers in post-dissection aneurysm subgroup. Further investigation is needed to better understand the peculiarity of this population.

In our cohort, all patients with AAD but one, who were discharged after FET operation were alive at last follow-up. In our view, Thoraflex Hybrid prosthesis facilitates successful surgical treatment for patients after such a high-risk event. The patients who survive early post-operative period are more likely to have uneventful midterm follow-up.

Our single-center experience shows the universal potential of this prosthesis for all kinds of aortic pathologies. Despite its initial purpose for distal aortic arch aneurysms, the international experience shows the versatile use of this prosthesis for all kinds of applications, such as penetrating aortic ulcers involving the aortic arch (21, 22). There are several reports where this prosthesis was even used upside down (reversed) for vascular surgery or post-dissection and thoracoabdominal aneurysm (23, 24).

Aortic arch prosthetic repair using an FET is also feasible in selected patients with AADB when the intimal tear is located directly beneath the left subclavian artery and there is no adequate landing length in Zone 2. It requires distal arch prosthetic repair with stentgrafting of the descending part of aorta, which is feasible on the beating heart.

A few limitations of this study should be outlined. It is retrospective by nature and limited to our single-center experience. Our results regrading low mortality and neurological deficits might be biased by the specialized team of surgeons.

Our experience demonstrates excellent perioperative results and, in our opinion, total aortic arch replacement with a Thoraflex Hybrid prosthesis is the most effective operative technique for type A aortic dissection. It causes rapid thrombosis of the false lumen and promotes a beneficial remodeling of the distal aorta, enabling first- and two-stage procedures. Next steps in our research will be further optimizing of perioperative management and extending follow-up for a more complete assessment and understanding of survival and freedom from reintervention.

## Data Availability Statement

The data analyzed in this study were not allowed to be made public according to the policy of our institute. Requests to access these datasets should be directed to konrad.wisniewski@ukmuenster.de.

## Ethics Statement

The studies involving human participants were reviewed and approved by Ethics Committee of the Medical Association of Westphalia-Lippe and the Faculty of Medicine, University of Münster (WWU Münster). Written informed consent for participation was not required for this study in accordance with the national legislation and the institutional requirements.

## Author Contributions

KW and AM were involved in the drafting of the manuscript. AD, AO, JS, AI, SM, and AR were involved in reviewing and providing feedback on the manuscript. All authors contributed to the article and approved the submitted version.

## Funding

This study was entirely funded by institutional funds of our department.

## Conflict of Interest

The authors declare that the research was conducted in the absence of any commercial or financial relationships that could be construed as a potential conflict of interest.

## Publisher's Note

All claims expressed in this article are solely those of the authors and do not necessarily represent those of their affiliated organizations, or those of the publisher, the editors and the reviewers. Any product that may be evaluated in this article, or claim that may be made by its manufacturer, is not guaranteed or endorsed by the publisher.

## Abbreviations

AADA: Acute Aortic Dissection Type A
AADB: Acute Aortic Dissection Type B
AAR: Ascending aortic replacement
CABG: Coronary artery bypass graft
CPB: Cardiopulmonary bypass
CPR: Cardiopulmonary resuscitation
CSF: Cerebrospinal fluid
CTA: Computed tomography angiography
ECLS: Extracorporeal life support
FET: Frozen elephant trunk
HCA: Hypothermic circulatory arrest
ICU: Intensive care unit
NIRS: Near-infrared spectroscopy
PDAA: Post-dissection aortic aneurysm
SACP: Selective antegrade cerebral perfusion
TAA: Thoracic aortic aneurysm.
